# Distribution and Habitat Associations of Billfish and Swordfish Larvae across Mesoscale Features in the Gulf of Mexico

**DOI:** 10.1371/journal.pone.0034180

**Published:** 2012-04-11

**Authors:** Jay R. Rooker, Jeff R. Simms, R. J. David Wells, Scott A. Holt, G. Joan Holt, John E. Graves, Nathan B. Furey

**Affiliations:** 1 Department of Marine Biology, Texas A&M University at Galveston, Galveston, Texas, United States of America; 2 Department of Wildlife and Fisheries Sciences, Texas A&M University, College Station, Texas, United States of America; 3 University of Texas Marine Science Institute, University of Texas at Austin, Port Aransas, Texas, United States of America; 4 Virginia Institute of Marine Science, College of William & Mary, Gloucester Point, Virginia, United States of America; Institute of Marine Research, Norway

## Abstract

Ichthyoplankton surveys were conducted in surface waters of the northern Gulf of Mexico (NGoM) over a three-year period (2006–2008) to determine the relative value of this region as early life habitat of sailfish (*Istiophorus platypterus*), blue marlin (*Makaira nigricans*), white marlin (*Kajikia albida*), and swordfish (*Xiphias gladius*). Sailfish were the dominant billfish collected in summer surveys, and larvae were present at 37.5% of the stations sampled. Blue marlin and white marlin larvae were present at 25.0% and 4.6% of the stations sampled, respectively, while swordfish occurred at 17.2% of the stations. Areas of peak production were detected and maximum density estimates for sailfish (22.09 larvae 1000 m^−2^) were significantly higher than the three other species: blue marlin (9.62 larvae 1000 m^−2^), white marlin (5.44 larvae 1000 m^−2^), and swordfish (4.67 larvae 1000 m^−2^). The distribution and abundance of billfish and swordfish larvae varied spatially and temporally, and several environmental variables (sea surface temperature, salinity, sea surface height, distance to the Loop Current, current velocity, water depth, and *Sargassum* biomass) were deemed to be influential variables in generalized additive models (GAMs). Mesoscale features in the NGoM affected the distribution and abundance of billfish and swordfish larvae, with densities typically higher in frontal zones or areas proximal to the Loop Current. Habitat suitability of all four species was strongly linked to physicochemical attributes of the water masses they inhabited, and observed abundance was higher in slope waters with lower sea surface temperature and higher salinity. Our results highlight the value of the NGoM as early life habitat of billfishes and swordfish, and represent valuable baseline data for evaluating anthropogenic effects (i.e., Deepwater Horizon oil spill) on the Atlantic billfish and swordfish populations.

## Introduction

Atlantic billfishes (family Istiophoridae) and swordfish (family Xiphiidae) are highly migratory species that frequent open ocean ecosystems throughout their ranges [1]–[3]. Populations of several Atlantic species, including blue marlin (*Makaira nigricans*), white marlin (*Kajikia albida*), sailfish (*Istiophorus platypterus*), and swordfish (*Xiphias gladius*), are assumed to be fully exploited or overfished with biomass of certain species below levels required to achieve maximum sustainable yield [4], [5]. Apart from their economic value in tropical and subtropical fisheries [6], billfishes and swordfish along with other oceanic predators play important roles in marine ecosystems [7]. Changes in abundances of oceanic predators can influence their top-down regulation of food webs, in some cases resulting in cascading effects that can alter the productivity, stability, and structure of marine ecosystems [8]–[10].

Conservation and rebuilding efforts for Atlantic billfish and swordfish stocks will ultimately rely on an improved understanding of habitats and environmental conditions required during ontogeny. Given that overexploitation and incidental bycatch of these oceanic predators are arguably the most critical issues facing fishery scientists [11], research on habitat use and movement of adult billfishes and swordfish is currently at the forefront because of the presumed value of these data for mitigating losses through the spatial management of fishing effort [12]–[15]. In contrast, our understanding of habitat use during the critical early life period is far less studied even though larval indices are valuable for identifying spawning/nursery grounds [16] and assessing population trends [17], [18]. In addition, it is well recognized that larval transport and/or survival varies as a function of time and location [19], [20], and therefore determining spatio-temporal patterns of habitat use during early life as well as identifying the biological and physicochemical attributes of presumed nurseries are needed to define essential habitats of Atlantic billfish and swordfish populations [21], [22].

Surveys of billfish and swordfish larvae in specific areas of the western North Atlantic Ocean (WNAO) and adjacent waters indicate that several regions are used as spawning and nursery areas of blue marlin, white marlin, sailfish, and swordfish. The majority of early life studies to date in the WNAO, particularly for billfishes, has occurred in a fairly restricted geographic area encompassing the Straits of Florida and the Bahamas [16], [23], [24], while data on the distribution and abundance of billfish and swordfish larvae from other potential spawning and nursery areas is presently limited or unavailable. The Gulf of Mexico is increasingly recognized as an important foraging and spawning area of Atlantic billfishes and swordfish, and this assumption is based primarily upon the fact that large numbers of adults are caught in this region by pelagic longliners during presumed spawning periods [25], [26]. As a result, spawning stock biomass within this region appears to be high, indicating that the Gulf may represent essential spawning and nursery habitat of these species.

The purpose of the present study was to conduct ichthyoplankton surveys in surface waters of the northern Gulf of Mexico (NGoM) over an extended time period to determine the relative value of this region as a spawning and nursery habitat of billfish and swordfish populations. In addition to documenting regional patterns of occurrence and abundance, we examined the influence of ocean conditions, both biotic and abiotic, on the distribution and abundance of blue marlin, white marlin, sailfish, and swordfish larvae using generalized additive models (GAMs). Outer shelf and slope waters of the NGoM represent an ideal location for this study because they are dominated by mesoscale features such as cyclonic and anti-cyclonic eddies and associated zones of confluence (i.e. fronts), which are assumed to be important early life habitats of pelagic fishes [27]. Moreover, the NGoM receives substantial freshwater inflow and nutrient loading from the Mississippi River and represents one of the most productive areas of the WNAO, adding to the unique nature of this region and its potential as important early life habitat of pelagic fishes. Finally, the NGoM was recently impacted by the Deepwater Horizon oil spill, and thus information included here represents valuable baseline data that can be used to evaluate the effects of this event on Atlantic billfish and swordfish populations.

## Methods

### Sampling design and data collection

**Figure 1:**
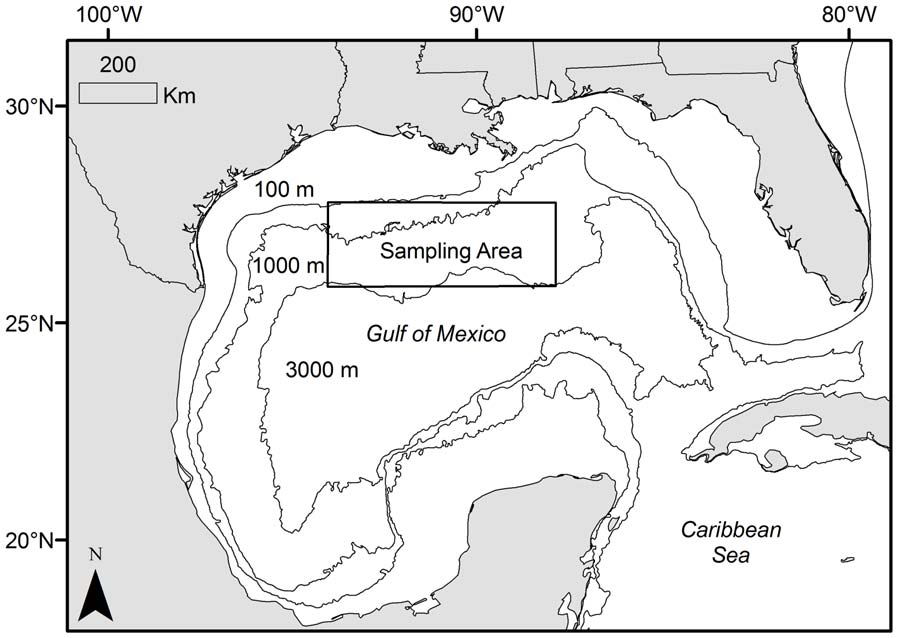
Map of sampling area (outlined) in outer shelf and slope waters of the northern Gulf of Mexico. Ichthyoplankton surveys conducted in June and July over the three-year period: 2006–2008.

Ichthyoplankton surveys were conducted in shelf and slope waters of the NGoM in a sampling corridor encompassing a region from approximately 26.5 to 28.0°N latitude and 88.0 to 94.0°W longitude (Fig. 1). Two surveys were conducted each year over the three-year study period (2006 to 2008), and sampling was restricted to June and July because this period represents the primary spawning months of targeted taxa [28]. All sampling was conducted during the day (ca. 0700 to 1900 h), and billfish and swordfish larvae were collected with paired neuston nets (2-m width ×1-m height frame) equipped with two different mesh sizes: 500 µm and 1200 µm. Nets were towed through surface waters at approximately 2.5 knots for 10 minutes, and paired tows were taken at each station. At the start of each tow, approximately 0.2 m of the neuston net frame was above the water; however, due to changes in sea state and a variety of other factors, the depth of the surface water sampled within and across net tows ranged from approximately 0.6 to 1.0 m. Sampling was conducted at approximately 15-km intervals between stations to allow coverage of large areas encompassing multiple oceanographic features. General Oceanics flowmeters (Model 2030R, Miami, FL) were placed within each neuston net to determine surface area sampled during each tow, which was used in conjunction with catch data to determine the abundance (i.e., density) of billfish and swordfish larvae at each sampling station. Permits for collections of fish larvae were issued by the Highly Migratory Species Management Division of the National Oceanic and Atmospheric Administration (permits: Billfish-SRP-06-01, Billfish-EFP-07-03, and Billfish-EFP-08-03).

Fishes and associated fauna collected with neuston nets were preserved onboard the vessel in 95% ethanol. *Sargassum* biomass (pooled weight of *S. natans* and S. *fluitans*) in each neuston net was also recorded. Ichthyoplankton were later sorted in the laboratory, and all billfish and swordfish larvae were separated from other taxa. Larvae were enumerated and the standard length (SL) of each individual was measured to the nearest 0.1 mm. Billfish and swordfish larvae do not have a full complement of fin rays until approximately 20 mm SL [29], [30]. Given that over 99% of the specimens collected with neuston nets were less than 20 mm SL, individuals are hereafter referred to as larvae. The abundance of blue marlin, white marlin, sailfish and swordfish larvae at each station was derived by summing catch numbers from both 500 and 1200 µm neuston nets and dividing by the distance towed of both nets combined. Because it is inherently difficult to determine the actual volume of water sampled with partially submerged neuston net deployments, abundance is reported here as the number of larvae collected per square meter of ‘surface water’ sampled. In response, our estimate of density is based on the number of larvae per unit area rather than volume. For all four species, density at each station was expressed as number of larvae 1000 m^−2^ of surface water, and average area sampled with both nets at each station was approximately 4500 m^2^.

Swordfish larvae were identified visually using anatomical and morphometric features [30]. Although diagnostic characters are evident for billfish larvae, species identification for smaller larvae (<10 mm SL) in this family is problematic and can lead to identification errors among the three species examined [24]. In response, identification of billfish larvae less than 10 mm SL was determined using a species-specific multiplex polymerase chain reaction (PCR) assay. Our multiplex PCR assay followed the protocol described by Simms et al. [24] and developed by J. Magnussen and M. Shivji at Nova Southeastern University. Briefly, a single eyeball was removed from each larva and DNA was extracted using a QIAGEN DNeasy blood and tissue kit. A multiplex PCR was performed using an Eppendorf mastercycler gradient, QIAGEN Hot Star Taq DNA Polymerase (QIAGEN # 203203), and PCR grade dNTP mix (QIAGEN # 201901). Four primer pairs were used in each PCR reaction: a universal billfish primer set and species-specific primers for sailfish, white marlin, and blue marlin. PCR products were examined by means of gel electrophoresis with 1% agarose gels containing ethidium bromide. Species identification was based on species-specific gel banding patterns visualized on an ultraviolet trans-illuminator. For the majority of our collections (>90%), all billfish larvae were assayed from individual net tows; however, for collections with more than 20 billfish larvae, a subsample of individuals was assayed and deemed sufficient for identification purposes unless the results indicated that more than one species of billfish was present.

Sea surface temperature and salinity were measured at each sampling station using a Sonde 6920 Environmental Monitoring System (YSI Inc.). Other environmental data at sampling stations were extracted from remotely sensed data to match sampling dates and locations. Sea surface height anomaly (SSHA, cm) and sea surface current velocity data (m s^−1^) were generated every 7 days from merged satellite altimetry measurements using Jason-1, ENVISAT/ERS, Geosat Follow-On and Topex/Poseidon interlaced (AVISO, www.aviso.oceanobs.com) [31]. Sea surface height anomaly and current velocity data consisted of averaged time periods with 0.25° resolution. Distance to the Loop Current was estimated by measuring the linear distance from the edge of this feature (based on the location of the 20-cm SSHA contour) to the sampling station. Sea surface chlorophyll concentrations (mg m^−3^) were downloaded from the Sea-viewing Wide Field-of-view Sensor (SeaWiFS) (http://las.pfeg.noaa.gov). Chlorophyll data consisted of 8-d averaged time periods with 0.1° resolution. Water depth at all sampling stations was extracted from Satellite Geodesy, Scripps Institution of Oceanography (http://topex.ucsd.edu/marine_topo/).

### Data analysis and modeling

Generalized additive models (GAMs) were used to investigate the influence of environmental conditions on the abundance (i.e., density) of each species. Density at each station was considered to be a count variable (non-negative integers) for modeling purposes. GAMs are a nonparametric extension of general linear models (GLMs) that allow for complex relationships between response and explanatory variables [32], [33]. General GAM construction followed the equation:
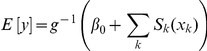
Where 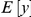 equals the expected values of the response variable (density), 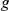 represents the link function, 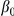 equals the intercept, 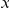 represents one of 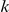 explanatory variables, and 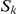 represents the smoothing function of each respective explanatory variable. Poisson models with a logarithm link were fit with cubic regression splines within the software R [34] *mgcv* library [35], [36]. The flexibility of splines are automatically reduced from a given maximum degrees of freedom by minimizing the General Cross Validation (GCV) score [35]. To avoid generating ecologically unrealistic responses (i.e. overfitting) [37], [38], cubic splines were restricted to 3 degrees of freedom (df). Lower and higher levels of variable complexity (2 and 4 df, respectively) were also examined and our conservative level of complexity (3 df) was deemed most appropriate given the tradeoff between model complexity and deviance [39].

A manual backwards stepwise procedure based on minimizing the Akaike Information Criterion (AIC, [40]) was used to select explanatory variables influencing larval density. Model selection was based on the premise that smaller AIC values represented models with the best fit, based on tradeoff between model complexity (number of variables) and fit (based on goodness of fit) [41]. Although backward selection was based on AIC values, approximate significance (p values) of smoothed variables in each model were also used to guide the backwards selection of variables. At the step when each of the remaining variables resulted in an increased AIC, the backward selection process was halted unless the removal of the non-significant term (p>0.05) resulted in an AIC value that was comparable (<1%) to that of the model that included this variable. Collinearity among explanatory variables was examined through Spearman rank correlation coefficient (Spearman ρ). When the Spearman ρ between two variables was greater than 0.5, the relative influence of each predictor was examined alone with a GAM and the variable that resulted in the lower AIC value was allowed to enter the initial model prior to backwards stepwise selection. Spatial autocorrelation was investigated for all four species by examining residuals from final GAMs with Moran's I; p-values were non significant (p>0.05) for all four species. To examine model fit, overall percent of deviance explained (DE) for each model was calculated (([null deviance – residual deviance]/null deviance) ×100). After final models were selected, we then excluded each variable individually from our final models and examined the change in both AIC or DE with and without each variable (denoted as ΔAIC and ΔDE), which provided a means of assessing the relative importance of each variable.

## Results

Overall, 2888 billfish and 264 swordfish larvae were collected during the six surveys conducted from 2006 to 2008 (Table 1). Sailfish larvae were the dominant billfish (n = 2033), accounting for 70.4% of all istiophorid larvae collected. Mean density of sailfish per survey ranged from 0.45 to 1.99 larvae 1000 m^−2^. Blue marlin (n = 722) and white marlin (n = 133) comprised 25.0% and 4.6% of the billfish larvae in our collections, respectively. Mean density of blue marlin per survey ranged from 0.04 to 0.76 larvae 1000 m^−2^, while white marlin ranged from 0.00 to 0.33 larvae 1000 m^−2^. Density of swordfish (n = 264) ranged from 0.04 to 0.32 larvae 1000 m^−2^ among the six surveys. Areas of peak production were detected, with maximum density estimates at single stations being markedly higher than survey means: sailfish (22.09 larvae 1000 m^−2^), blue marlin (9.62 larvae 1000 m^−2^), white marlin (5.44 larvae 1000 m^−2^), and swordfish (4.67 larvae 1000 m^−2^).

**Table 1:** Summary data on collection of billfish and swordfish larvae for six surveys conducted from 2006 to 2008 in the northern Gulf of Mexico.

	Sailfish			Blue marlin			White marlin			Swordfish		
Survey date	N	Density	% Freq	N	Density	% Freq	N	Density	% Freq	N	Density	% Freq
June 15–20, 2006	691	1.99	48.4	18	0.06	16.1	22	0.06	16.1	20	0.06	17.7
July 31-Aug 6, 2006	634	1.71	47.7	346	0.43	33.8	1	0.00	1.5	136	0.33	29.2
June 20–24, 2007	213	0.45	23.7	102	0.31	27.1	30	0.09	15.3	59	0.15	20.3
July 20–24, 2007	70	0.42	32.8	182	0.76	41.4	0	0.00	0.0	14	0.07	15.5
June 9–13, 2008	259	0.84	37.3	12	0.04	8.0	78	0.30	14.7	23	0.08	10.7
July 27-Aug 1, 2008	166	0.37	24.1	62	0.22	24.1	2	0.01	2.4	12	0.04	9.6
	**2033**	**0.96**	**35.7**	**722**	**0.30**	**25.1**	**133**	**0.08**	**8.3**	**264**	**0.12**	**17.2**

Count (N), density (larvae 1000 m^−2^), and percent frequency of occurrence estimates are shown for sailfish, blue marlin, white marlin and swordfish.

Intra- and/or inter-annual variation in the occurrence and abundance of larvae was detected for all four species (Table 1). Sailfish larvae were detected in all months and years sampled, with percent frequency of occurrence per survey (based on presence at stations sampled) ranging from 24.8% (July 2008) to 48.4% (July 2006). Surveys with the highest mean density of sailfish were June 2006 (1.99 larvae 1000 m^−2^) and July 2006 (1.71 larvae 1000 m^−2^), and mean density was lower for 2007 and 2008 surveys (range 0.36 to 0.84 larvae 1000 m^−2^). Blue marlin larvae were collected in all surveys but density was higher each year during the July survey: 2006 (0.43 larvae 1000 m^−2^), 2007 (0.76 larvae 1000 m^−2^), and 2008 (0.21 larvae 1000 m^−2^). Percent frequency of occurrence of blue marlin larvae among surveys was also higher in July (24.1 to 33.8% across years) than June (8.0 to 27.1% across years). Density of white marlin larvae peaked during June surveys, with the highest overall density occurring in June 2008 (0.30 larvae 1000 m^−2^). Percent frequency of occurrence of white marlin during June surveys ranged from 14.7 to 16.1%, which is nearly an order of magnitude higher than values observed for July surveys (range 0.0 to 2.4%). Swordfish larvae were collected in all months and years surveyed, and mean density was greatest for the July 2006 survey (0.32 larvae 1000 m^−2^). Percent frequency of occurrence for swordfish ranged from 9.6% (July 2008) to 29.2% (July 2006).

**Figure 2:**
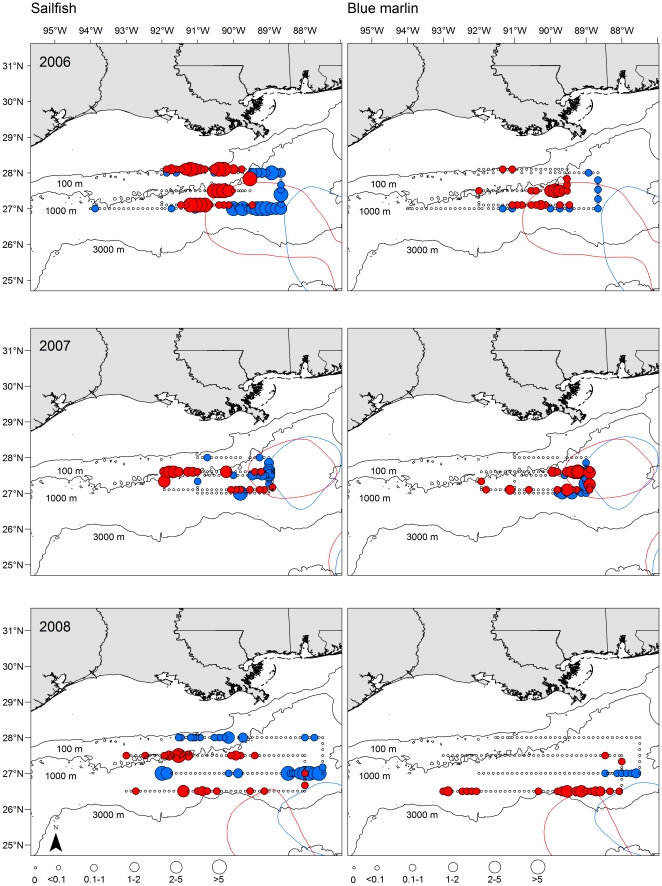
Spatial and temporal (month and year) variability in the density of sailfish and blue marlin larvae collected in ichthyoplankton surveys from 2006 (top), 2007 (middle), and 2008 (bottom) in the northern Gulf of Mexico. June (blue) and July (red) survey shown and colored lines represent the observed margin of the Loop Current during each sampling trip (coded by color). Density (larvae 1000 m^−2^) denoted by circle size.

**Figure 3:**
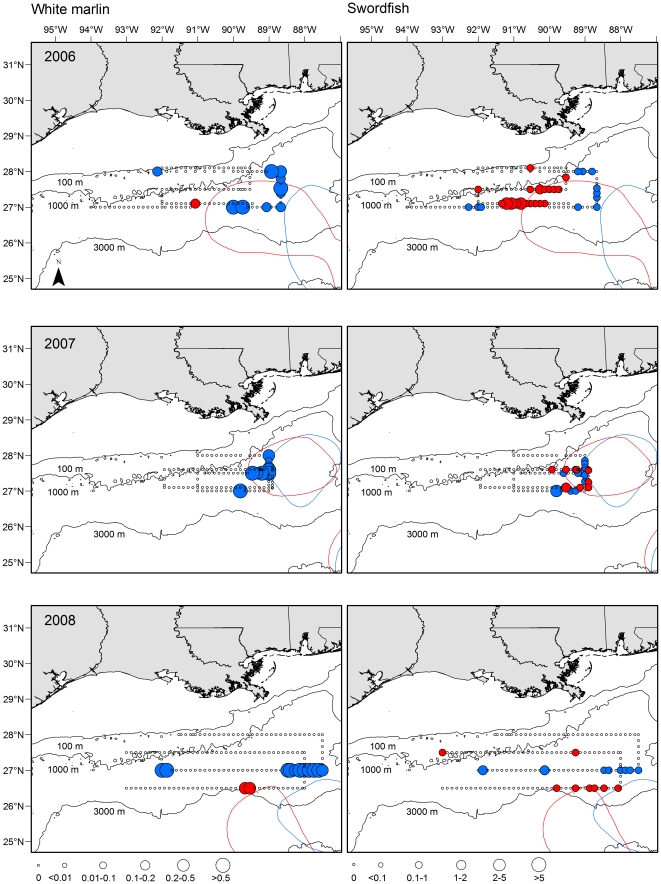
Spatial and temporal (month and year) variability in the density of white marlin and swordfish larvae collected in ichthyoplankton surveys from 2006 (top), 2007 (middle), and 2008 (bottom) in the northern Gulf of Mexico. June (blue) and July (red) survey shown and colored lines represent the observed margin of the Loop Current during each sampling trip (coded by color). Density (larvae 1000 m^−2^) denoted by circle size.

Longitudinal and latitudinal patterns in the distribution and abundance of billfish and swordfish larvae were detected, and general spatial trends were consistent across survey years for certain species. Of the four taxa examined, sailfish were the most widespread (Fig. 2), with individuals frequently found throughout much of our sampling corridor in the majority of surveys. Nevertheless, some degree of longitudinal variation was present for sailfish with peak catches typically east of 91°W in June and shifting more westward in July. For blue marlin, distributions were more restricted and larvae were primarily collected in areas east of 90°W, particularly in areas proximal to the western margin of the Loop Current in waters south of 28°N (Fig. 2). Similarly, white marlin larvae were caught predominantly in waters east of 90°W in all three years, and the occurrence and abundance were typically highest along the western margin of the Loop Current in slope waters located at approximately 88°W (Fig. 3). East to west variation in swordfish density was less pronounced, although catches in certain years (i.e., 2007) were higher east of 90°W in both outer shelf (∼28°N transect) and slope waters south of the shelf break (Fig. 3). Several areas of high density were detected for swordfish, with most larvae in areas between 89°W and 91.5°W.

### Final GAMs and model performance

Initial correlation analysis was performed for oceanographic and position variables (Table S1). Significant collinearity (Spearman ρ>0.5) was detected between latitude and water depth (0.71), longitude and water depth (0.52), and longitude and distance to the Loop Current (0.72). The relative influence of each variable was examined with GAMs and lower AIC scores were observed for models that included water depth and distance to the Loop Current over those with latitude or longitude. In response, latitude and longitude were excluded as variables in the backwards selection process for determining the final models of each species. Final models were based on abundance (density) data due the high explanatory power of these models relative to GAMs based on presence/absence data (Table S2).

### Sailfish model

**Figure 4:**
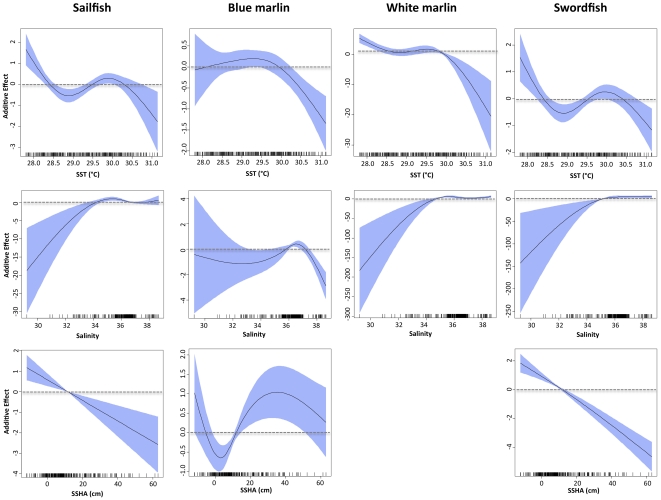
Response plots for abiotic variables on the density of sailfish, blue marlin, white marlin, and swordfish larvae from final generalized additive models (GAMs). Plot includes sea surface temperature (top), salinity (middle) and sea surface height anomaly (bottom). Solid lines denote smoothed values and shaded areas on each plot represent 95% confidence intervals. Dashed line at y = 0 displayed on each plot.

**Figure 5:**
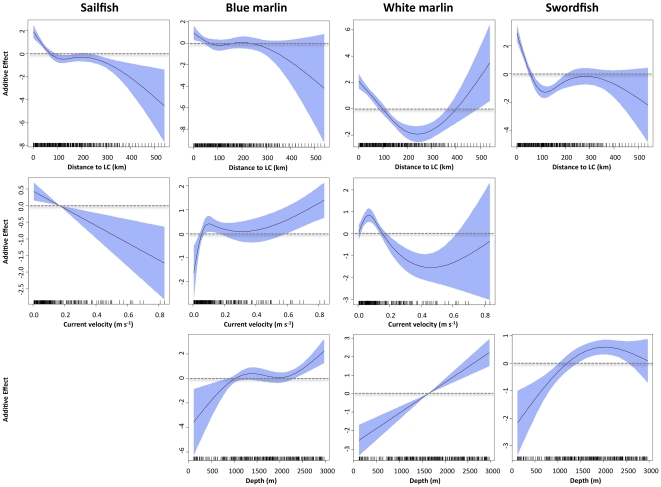
Response plots for abiotic variables on the density of sailfish, blue marlin, white marlin, and swordfish larvae from final generalized additive models (GAMs). Plot includes distance to Loop Current (top), sea surface current velocity (middle) and depth (bottom). Solid lines denote smoothed values and shaded areas on each plot represent 95% confidence intervals. Dashed line at y = 0 displayed on each plot.

**Figure 6:**
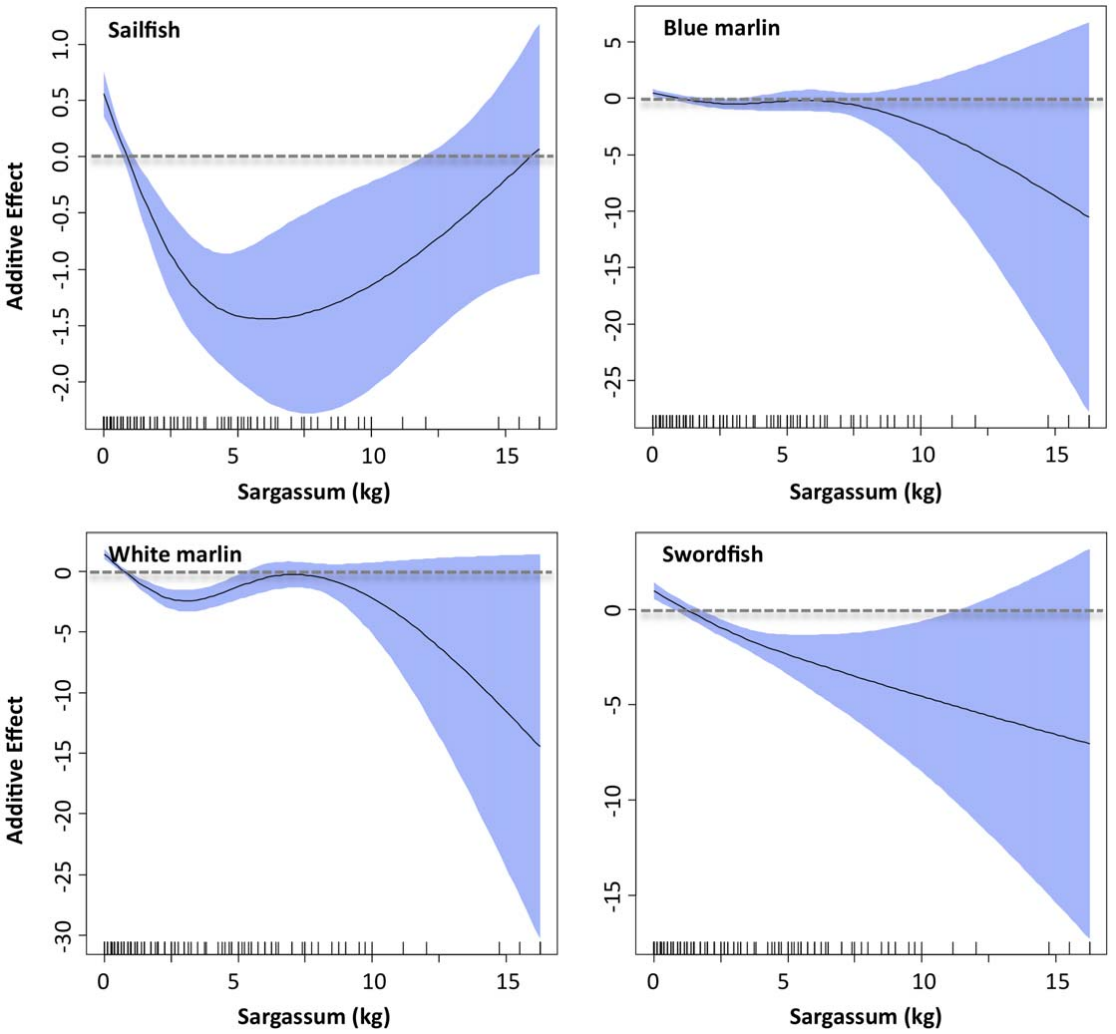
Response plots of biotic variable, *Sargassum* biomass, on the density of sailfish, blue marlin, white marlin, and swordfish larvae from final generalized additive models. Solid lines denote smoothed values and shaded areas on each plot represent 95% confidence intervals. Dashed line at y = 0 displayed on each plot.

The final sailfish model included 7 variables, and AIC for the final model was 8407, with percent of deviance explained at 43.6% (Table 2). Retained variables with associated ΔAIC values were distance to Loop Current (1283), *Sargassum* (825), year (580), sea surface temperature (473), salinity (386), sea surface height anomaly (326), and sea surface current velocity (223) (Table 2). ΔDE was also used to assess the importance of each environmental variable for the sailfish model, and the relative contribution of retained variables was similar between the two measures. Response plots from the sailfish GAM indicated that density of sailfish larvae was higher in waters close to the Loop Current (<100 km) with moderate to high salinity (>34), low to moderate sea surface temperatures (<30.5°C), and in areas with negative (cold core eddies) or slightly positive sea surface height anomalies (Fig. 4, 5). Sailfish larvae were more abundant in areas with both high and low *Sargassum* biomass compared to stations with moderate levels (5 to 25 kg) (Fig. 6). Variables not retained in the sailfish model were month, sea surface chlorophyll, and water depth.

**Table 2:** Environmental and temporal variables in final generalized additive models for sailfish, blue marlin, white marlin and swordfish.

	Sailfish AIC =	8407	DE = 43.6%	Blue marlin AIC =	2140	DE = 62.3%
Variable	Final Model	Delta AIC	Delta DE	Final Model	Delta AIC	Delta DE
**Month**						
**Year**	Year	580	3.8%	Year	201	4.5%
						
**SSHA**	SSHA	326	2.3%	SSHA	93	2.1%
**SSCV**	SSCV	223	1.6%	SSCV	97	2.2%
**SSChl**						
**SST**	SST	473	1.4%	SST	113	2.6%
**Salinity**	Salinity	386	2.8%	Salinity	256	5.8%
**Water Depth**				Depth	142	3.3%
**Sargassum**	Sargassum	825	6.0%	Sargassum	162	3.8%
**Distance LC**	Distance LC	1283	9.3%	Distance LC	42	1.0%

	White marlin AIC =	570	DE = 79.6%	Swordfish AIC	1174	DE = 55.9%
Variable	Final Model	Delta AIC	Delta DE	Final Model	Delta AIC	Delta DE
**Month**	Month	17	0.9%			
**Year**	Year	13	0.9%	Year	142	7.2%
**SSHA**				SSHA	235	12.0%
**SSCV**	SSCV	32	1.4%			
**SSChl**	SSChl	35	1.6%			
**SST**	SST	99	5.1%	SST	37	2.1%
**Salinity**	Salinity	58	3.3%	Salinity	79	4.3%
**Water Depth**	Depth	76	2.4%	Depth	69	3.7%
**Sargassum**	Sargassum	126	4.6%	Sargassum	155	7.8%
**Distance LC**	Distance LC	91	4.5%	Distance LC	242	12.0%

Akaike's Information Criterion (AIC) and percent deviance explained (DE) is given for each final model. ΔAIC and ΔDE values are based on the difference if the variable was excluded from the final model. Variables (codes): sea surface height anomaly (SSHA), sea surface current velocity (SSCV), sea surface chlorophyll (SSChl), sea surface temperature (SST), and Distance to Loop Current (distance LC).

### Blue marlin model

The final blue marlin model included 8 variables, and AIC for the final model was 2140, with percent of deviance explained at 62.3% (Table 2). ΔAIC values of retained variables indicated that sea surface salinity (256), year (201), *Sargassum* biomass (162), and water depth (142) were the most influential variables, with sea surface temperature (113), sea surface current velocity (97), sea surface height anomaly (93), and distance to Loop Current (42) also contributing. ΔDE for variables retained in the model ranged from 1.0 to 5.8%, and similar to ΔAIC, observed ΔDE values were the highest for sea surface salinity (5.8%), year (4.5%), and *Sargassum* (3.8%). Response plots from the blue marlin GAM showed that larvae were most abundant in areas closer to the Loop Current and in surface waters with higher current velocities relative to other areas sampled (Fig. 4, 5). In addition, density was positively related to water depth and greater in areas with lower sea surface temperatures (28–30°C) and higher salinity (36 to 37) (Fig. 4), which are conditions typically associated with oceanic water masses off the continental shelf in slope waters. Density of blue marlin larvae was higher in areas with both negative and positive sea surface height anomalies (Fig. 4), suggesting that larvae were associated with cold and warm cores features. Finally, density of blue marlin larvae was inversely related to *Sargassum* biomass collected in neuston nets (Fig. 6).

### White marlin model

The final white marlin model included 9 variables, and AIC for the final model was 570, with percent of deviance explained at nearly 80% (Table 2). Influential variables with associated ΔAIC values were *Sargassum* biomass (126), sea surface temperature (99), distance to Loop Current (91), water depth (76), and sea surface salinity (58); ΔDE for these influential variables ranged from 2.4 to 5.1% (Table 2). ΔAIC values for the four remaining variables included in the final model (sea surface chlorophyll, sea surface current velocity, month, year) were less than 40, and ΔDE was less than 2% for each of these variables. Response plots from the white marlin GAM indicated that larvae were most abundant in areas with lower sea surface temperatures (28–30°C) relative to other areas surveyed (Fig. 4). In addition, density was positively related to both water depth and sea surface salinity (Fig. 4, 5), suggesting that larvae were most abundant in areas off the continental shelf (i.e., slope waters) or rather areas far removed from coastal influences such as freshwater inflow from the Mississippi River. Relationships with distance to Loop Current and sea surface current velocity were more complex with the density of white marlin larvae greater at both the lower and higher ends of the ranges for both variables (Fig. 5). Similar to blue marlin, density was lowest in collections with large amounts of *Sargassum* (Fig. 6).

### Swordfish model

The final swordfish model included 7 variables, and AIC for the final model was 1174, with percent of deviance explained at 54.9% (Table 2). Retained variables with associated ΔAIC values were distance to Loop Current (242), sea surface height anomaly (235), *Sargassum* biomass (155), year (142), sea surface salinity (79), water depth (69), and sea surface temperature (37). The AIC for the final model was 1174, and percent of deviance explained by this model was 55.9%. The relative importance of each variable as indicated by ΔDE was consistent with ΔAIC; ΔDE of the three most influential variables (distance to Loop Current, sea surface height anomaly, and *Sargassum*) were between 7.8 and 12.0% (Table 2). Response plots from the swordfish GAM showed that larvae were most abundant in areas with moderate to high salinity (34–38) and low sea surface temperature (<28.5°C), relative to other areas surveyed (Fig. 4). Similar to sailfish, a negative relationship between sea surface height anomaly and swordfish density was observed (Fig. 4), with the highest abundances in areas with negative anomalies (i.e., cold core eddies). Density was highest near the Loop Current, particularly at water depths from 1500–2500 m (Fig. 5). Density of swordfish larvae was negatively related to *Sargassum* biomass, with high catches at stations with little or no *Sargassum* (Fig. 6).

## Discussion

The occurrence and density of billfish and swordfish larvae in our study area indicate that the NGoM serves as spawning and early life habitat of all four species, particularly sailfish and blue marlin. Direct comparisons of abundance with other studies are problematic due to differences in sampling designs and gear types; nevertheless, our estimates of mean and maximum densities as well as percent frequency of occurrence of sailfish and blue marlin larvae were comparable or at times higher than reported values from other putative spawning and nursery areas in the western Atlantic, including the Straits of Florida and the Bahamas [23], [42], [43]. Over the three-year sampling period, nearly 40% of our net tows contained sailfish larvae with blue marlin present in approximately 25% of the samples. Both species were consistently caught over the three-year study period and present during all surveys. In addition, peak densities of sailfish (22 larvae 1000 m^−2^) and blue marlin (10 larvae 1000 m^−2^) were at the upper end of previously reported ranges for these species [23], [26], [42]. Although swordfish and white marlin catches were lower, white marlin larvae were present in all but one survey while swordfish larvae were present in all surveys, indicating that the region may represent suitable spawning and nursery habitat for some fraction of the Atlantic populations of both species.

Intra- and inter-annual variability in the occurrence or abundance of larvae is common for pelagic species inhabiting the NGoM [22], [44]. Given that our sampling was limited to the mid-summer period of June and July, the lack of a month or intra-annual effect was not unexpected for sailfish and blue marlin, which are known to spawn throughout the summer [28], [43]. Swordfish display year-round spawning in the Atlantic Ocean [43], [45], [46], and similar to sailfish and blue marlin, no month effect was detected. Conversely, month was retained as a variable in the white marlin model, which was likely due to the fact that this species typically spawns from April to June [47]. Therefore, our July sampling period was outside the primary spawning season of white marlin, explaining the low catch numbers observed during this month. Inter-annual variability in the occurrence and abundance of each species was more pronounced and varied markedly over the three-year sampling period. In fact, maximum observed density among the six surveys occurred in different years for the three billfish species: sailfish (June 2006), blue marlin (July 2007), and white marlin (June 2008). Temporal variability in egg and larval production has been linked to differences in the abundance (spawning stock biomass) and demographics (age structure) of adults [48]–[50], and thus annual shifts in the spawning stock biomass of all four species in the NGoM may be partly responsible for observed inter-annual trends in our catch numbers of larvae.

**Figure 7:**
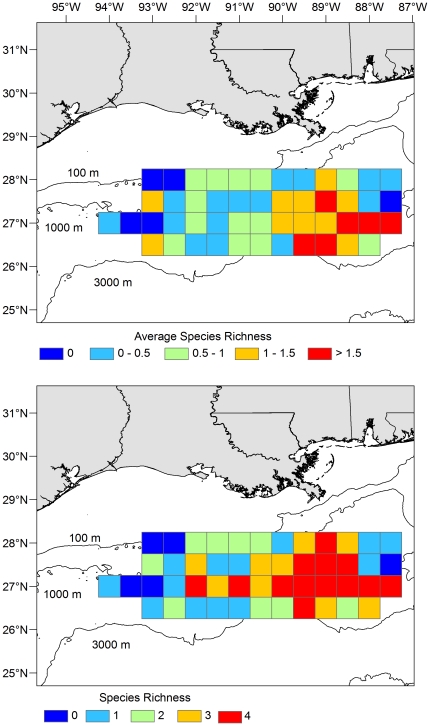
Diversity of billfish and swordfish catches in northern Gulf of Mexico. Average species richness (top) based on the mean number of target species (sailfish, blue marlin, white marlin, and swordfish) collected during each net deployment. Species richness (bottom) based on the total number of target species observed in each area. Cell size for estimates of diversity set at 0.25° and samples were pooled across the three-year sampling period (2006–2008).

Similar associations between larval density and the abiotic or biotic factors included in our GAMs were observed for sailfish, blue marlin, white marlin, and swordfish. In fact, response plots (smoothing curves) depicting relationships between larval density and environmental variables (e.g., sea surface temperature, salinity, distance to Loop Current, water depth, *Sargassum* biomass) were often similar for three or four of the species examined. Analogous habitat requirements convey that some degree of niche overlap occurs and emphasizes the importance of outer continental shelf and slope waters as early life habitat of all four species. In addition, our results indicate that slope waters in the eastern section of our sampling corridor represent a hotspot for billfishes and swordfish (Figure 7), and therefore future efforts to rebuild Atlantic stocks of these pelagic fishes may benefit by protecting this specific region of the NGoM.

While the importance of biotic and abiotic factors was often similar between and among species examined, differences in the percent deviance explained (DE) for each final model provided species-specific insights regarding what constitutes important or suitable early life habitat of each species. We observed that percent deviance explained in final models of the blue marlin and white marlin was considerably higher than the sailfish model and to a lesser degree swordfish. Interestingly, sailfish larvae were the most abundant, and the horizontal distribution of this species was far more widespread than the other three taxa, signifying that sailfish larvae are present across a wider range of environmental conditions compared to the three other species. In contrast, the more limited distribution of blue marlin and white marlin is possibly due to increased sensitivity or restricted tolerances to environmental conditions. The reduced ‘capture’ windows or limited distributions of blue marlin and white marlin likely increased the relative influence or strength of predictor variables in the final models, which in turn improved overall fits of these models relative to the sailfish model. Similarly, other studies have demonstrated that the predictive capabilities of habitat suitability models for species or populations with smaller distributions/ranges or more limited physiological tolerances to environmental conditions are typically superior to species that range widely [38], [51].

Physicochemical conditions within a nursery area are known to influence survival and cohort biomass, particularly salinity and temperature, which have been shown to be important variables in habitat suitability models for early life stages of marine fishes [52]–[54]. In the present study, salinity and temperature were each retained in the final models of all four species, and both appear to be important determinants of habitat quality for these species. Density of each species was typically greater at higher salinities and lower temperatures within the range observed in our sampling corridor. These conditions correspond to offshore water masses in the NGoM, and thus it is not surprising that water depth was retained in the final models of blue marlin, white marlin, and swordfish, with larval density of the three taxa positively associated with water depth. Such observations imply that stable water masses off the continental shelf (i.e., slope waters) represent more suitable habitat of billfish and swordfish larvae. Coastal water masses in the NGoM are influenced to a large degree by freshwater inflow from the Mississippi River, and tongues of lower salinity water with unique salinity-temperature profiles and color (i.e., green) were transported to stations within our sampling corridor on several occasions. Billfish and swordfish larvae were rare at stations inundated by coastally derived water masses that were characterized by lower salinity and higher sea surface temperature. Given that most pelagic larvae are highly sensitive to changes in salinity and/or temperature [55], [56], areas where coastal and offshore water masses mix may maintain physicochemical conditions that are unfavorable physiologically to billfish and swordfish larvae, possibly resulting in lower condition, lower growth, and higher mortality compared to individuals transported to or entrained in offshore water masses.

Mesoscale hydrographic features within ocean basins are also known to influence the transport and retention of larvae through physical processes, which in turn regulate patterns of distribution and abundance [57]–[59]. In the NGoM, the Loop Current is the most conspicuous mesoscale feature and areas of convergence between this current and associated features (eddies) are often associated with increased primary and secondary production [60], [61]. In the present study, abundances of sailfish, blue marlin, and swordfish larvae increased as distance to the Loop Current declined, with numbers peaking at stations near or on the margin of this boundary current. This finding supports the premise that billfish and swordfish larvae aggregate on or close to frontal features near the periphery of the Loop Current. The entrainment or aggregation of fish larvae at frontal zones with sharp gradients in physicochemical properties is well documented, and the abundance of larvae is routinely higher in frontal zones relative to adjacent water masses [62], [63]. The accumulation of fish larvae within frontal features is primarily attributed to the physical process of hydrodynamic convergence [64], and the complexity and stability of convergence zones often determines the level of retention or dispersion of larvae within these areas of discontinuity [65]. If observed aggregations of billfish and swordfish larvae along the frontal zone of the Loop Current is primarily due to hydrodynamic convergence, then intra- and inter-annual variation in the spatial configuration (shape and penetration) of this mesocale feature and associated frontal zones may determine the spatial distribution of billfish and swordfish larvae in the NGoM.

Fronts, eddies, and associated areas of convergence are also known to enhance the vertical transport of nutrients to surface waters, often resulting in higher primary and secondary production relative to surrounding areas [66]. In response, ecological conditions at frontal zones, namely enhanced prey resources, may have improved the growth and survival of billfish and swordfish larvae, possibly leading to higher overall abundances. Evidence of a ‘trophic advantage’ due to increased production has been demonstrated previously for species inhabiting frontal zones (e.g., improved condition, growth, and survival [67]). In our study, sea surface height anomaly was retained in the final models of sailfish, blue marlin, and swordfish, and we observed higher densities of all three taxa at stations with negative sea surface height anomalies. The presence of negative sea surface height anomalies often signifies that upwelling is occurring, which results in a flux of cool, nutrient-rich waters into the euphotic zone. Eddy-induced upwelling is an important source of new production in the Atlantic Ocean and Gulf of Mexico, and has been shown to increase overall biological productivity in pelagic ecosystems [68], [69]. Our observation of peak densities of billfish and swordfish larvae in regions with negative sea surface height anomalies and lower sea surface temperatures suggest that these areas could be experiencing local upwelling and enhanced production. In turn, new production may enhance prey resources for consumers such as billfish and swordfish larvae, potentially resulting in improved survival rates and higher cohort biomass. Although elevated abundances of billfish and swordfish larvae in areas with negative sea surface height anomalies may be due to a trophic advantage, sea surface chlorophyll (proxy for primary production) was only retained in one of the models (white marlin) and its influence was minor.

In addition to primary production by phytoplankton, *Sargassum* is another producer known to influence the distribution and abundance of pelagic consumers, often aggregating juvenile fishes [70], [71]. *Sargassum* biomass was inversely related to the abundance of blue marlin, white marlin, and swordfish larvae, indicating that surface waters with significant amounts of *Sargassum* may not represent suitable habitat for billfish and swordfish larvae. Our finding of a negative relationship with *Sargassum* biomass may be due in part to the fact that these floating mats concentrate juvenile fishes or potential predators of billfish and swordfish larvae [72], which would result in higher predation mortality and lower overall abundance. Previous research in the NGoM has shown that natural mortality rates on pelagic fish larvae can be higher in areas that concentrate predators (e.g. Mississippi River plume or associated fronts) relative to other areas of the continental shelf [67], and therefore increased predator biomass under floating *Sargassum* mats may correspondingly lead to higher natural mortality rates for billfish and swordfish larvae. Alternatively, the negative relationship between *Sargassum* and the abundance of billfish and swordfish larvae may be due to water mass properties and the geographic location of our stations. The production and biomass of *Sargassum* is known to increase markedly as mats move into nearshore waters with higher nutrient loads, and our net tows with the greatest *Sargassum* biomass were typically observed at northern stations (28°N) or areas closer to freshwater sources. The mismatch between *Sargassum* biomass and the density of billfish or swordfish larvae may be due to the fact that environmental conditions and regions that promote *Sargassum* growth are simply unfavorable for billfish and swordfish larvae. Interestingly, the only species showing increases in abundance at moderate to high *Sargassum* biomass was sailfish, and adults of this species often reside in more coastal regions relative to blue marlin, white marlin, or swordfish [73], [74]. Regardless of the mechanism responsible for the observed mismatch, our results clearly show that unlike other species that rely on *Sargassum* as essential fish habitat [70], [72], it does not appear to represent important early life habitat of blue marlin, white marlin or swordfish, at least during the first few weeks of life.

### Conclusions

Fisheries-independent indices of Atlantic billfish and swordfish abundance are valuable for assessing managed stocks, and larval-based indices are increasingly being used to assess population trends and identify critical spawning and nursery areas. Here, we highlight the value of the NGoM as important early life habitat of billfishes and swordfish, and describe specific associations between billfish and swordfish larvae and oceanographic conditions using a GAM framework. Our results clearly demonstrate that mesoscale features impact the distribution and abundance of billfish and swordfish larvae, and habitat suitability of all four species appears to be strongly linked to physicochemical attributes of the water masses they inhabit.

## Supporting Information

Table S1 **Description of environmental and position variables included in generalized additive models for sailfish, blue marlin, white marlin, and swordfish.** Mean and standard deviation associated with each parameter provided for each year of the study.
(DOCX)

Table S2 **Final binomial generalized additive models for sailfish, blue marlin, white marlin and swordfish based on presence/absence data.** Backward stepwise selection based on minimizing AIC was used to select a final model for each species. Akaike's Information Criterion (AIC) and percent deviance explained (DE) is given for each final model. ΔAIC and ΔDE values are based on difference if the variable was excluded from the final model.
(DOCX)
