# Supplementary material for: Distribution and Habitat Associations of Billfish and Swordfish Larvae across Mesoscale Features in the Gulf of Mexico
Source: PLoS One. 2012 Apr 11;7(4):e34180. doi: 10.1371/journal.pone.0034180 (PMC3324529; doi:10.1371/journal.pone.0034180)
Supplement: Table S1 — Description of environmental and position variables included in generalized additive models for sailfish, blue marlin, white marlin, and swordfish. Mean and standard deviation associated with each parameter provided for each year of the study. (DOCX) [file pone.0034180.s001.docx]

|  | **2006** | **2007** | **2008** |
| --- | --- | --- | --- |
|  | **Mean (SD)** | **Mean (SD)** | **Mean (SD)** |
| **Variable** |  |  |  |
| Water depth (m) | 1319 (713) | 1606 (586) | 1899 (809) |
| Latitude (°N) | 27.44 (0.42) | 27.38 (0.34) | 27.22 (0.54) |
| Longitude (°W) | -90.79 (1.32) | -90.07 (0.88) | -89.92 (1.53) |
| Salinity | 36.00 (0.47) | 36.67 (0.96) | 35.81 (1.54) |
| Sargassum biomass (kg) | 4.98 (7.11) | 2.20 (3.90) | 2.02 (4.80) |
| Sea surface chlorophyll, SSChl (mg m^-3^) | 0.11 (0.03) | 0.13 (0.05) | 0.22 (0.39) |
| Sea surface height anomaly, SSHA (cm) | 15.14 (15.72) | 9.76 (13.03) | 9.50 (11.97) |
| Sea surface temperature, SST (°C) | 29.37 (0.81) | 29.59 (0.84) | 29.01 90.67) |
| Sea surface current velocity, SSCV (m s^-1^) | 0.20 (0.22) | 0.17 (0.16) | 0.14 (0.11) |
| Distance to Loop Current (km) | 145 (151) | 90 (75) | 162 (95) |
