# Supplementary material for: Distribution and Habitat Associations of Billfish and Swordfish Larvae across Mesoscale Features in the Gulf of Mexico
Source: PLoS One. 2012 Apr 11;7(4):e34180. doi: 10.1371/journal.pone.0034180 (PMC3324529; doi:10.1371/journal.pone.0034180)
Supplement: Table S2 — Final binomial generalized additive models for sailfish, blue marlin, white marlin and swordfish based on presence/absence data. Backward stepwise selection based on minimizing AIC was used to select a final model for each species. Akaike's Information Criterion (AIC) and percent deviance explained (DE) is given for each final model. ΔAIC and ΔDE values are based on difference if the variable was excluded from the final model. (DOCX) [file pone.0034180.s002.docx]

|  |  | **Sailfish AIC =** | **458** | **DE=18.2%** |  | **Blue marlin AIC =** | **301** | **DE= 37.0%** |
| --- | --- | --- | --- | --- | --- | --- | --- | --- |
| **Variable** |  | **Final Model** | **Delta AIC** | **Delta DE** |  | **Final Model** | **Delta AIC** | **Delta DE** |
| **Month** |  |  |  |  |  | Month | 11 | 2.6% |
| **Year** |  | Year | 10 | 3.9% |  | Year | 8 | 1.8% |
|  |  |  |  |  |  |  |  |  |
| **SSHA** |  | SSHA | 6 | 2.8% |  |  |  |  |
| **SSCV** |  |  |  |  |  |  |  |  |
| **SSChl** |  |  |  |  |  |  |  |  |
| **SST** |  |  |  |  |  | SST | 8 | 2.3% |
| **Salinity** |  | Salinity | 12 | 3.3% |  |  |  |  |
| **Water Depth** |  | Depth | 2 | 1.3% |  | Depth | 20 | 5.0% |
| **Sargassum** |  | Sargassum | 3 | 2.0% |  | Sargassum | 4 | 0.4% |
| **Distance LC** |  | Distance LC | 20 | 5.5% |  | Distance LC | 52 | 13.0% |
|  |  |  |  |  |  |  |  |  |
|  |  |  |  |  |  |  |  |  |
|  |  | **White marlin AIC =** | **141** | **DE=52.3%** |  | **Swordfish AIC** | **252** | **DE=38.5%** |
| **Variable** |  | **Final Model** | **Delta AIC** | **Delta DE** |  | **Final Model** | **Delta AIC** | **Delta DE** |
| **Month** |  |  |  |  |  |  |  |  |
| **Year** |  |  |  |  |  | Year | 12 | 4.8% |
|  |  |  |  |  |  |  |  |  |
| **SSHA** |  | SSHA | 3 | 2.1% |  | SSHA | 13 | 5.7% |
| **SSCV** |  |  |  |  |  |  |  |  |
| **SSChl** |  |  |  |  |  |  |  |  |
| **SST** |  | SST | 19 | 10.5% |  |  |  |  |
| **Salinity** |  | Salinity | 20 | 10.4% |  | Salinity | 3 | 2.1% |
| **Water Depth** |  | Depth | 4 | 2.4% |  | Depth | 3 | 1.8% |
| **Sargassum** |  | Sargassum | 18 | 10.7% |  | Sargassum | 19 | 7.3% |
| **Distance LC** |  | Distance LC | 8 | 5.2% |  | Distance LC | 30 | 10.6% |
|  |  |  |  |  |  |  |  |  |
